# Short-Chain Mono-Alkyl β-D-Glucoside Crystals—Do They Form a Cubic Crystal Structure?

**DOI:** 10.3390/molecules27144359

**Published:** 2022-07-07

**Authors:** Shigesaburo Ogawa, Isao Takahashi

**Affiliations:** 1 Department of Food, Aroma and Cosmetic Chemistry, Faculty of Bio-Industry, Tokyo University of Agriculture, 196 Yasaka, Hokkaido 099-2493, Japan; 2 Department of Physics, School of Science and Technology, Kwansei Gakuin University, Sanda 669-1337, Japan

**Keywords:** alkyl glucoside, crystal, hydrate, cubic phase, powder X-ray diffraction (PXRD)

## Abstract

Three-dimensional liquid crystal (LC) phases, cubic LC phases, have been extensively studied as fascinating molecular assembled systems formed by amphiphilic compounds. However, similar structures have only been seen in rare instances in lipid crystal states in glycolipid crystal studies. In this study, we prepared short-chain n-alkyl β-D-glucosides (CnG) with an alkyl chain length *n* ranging from 4 to 6 and investigated their crystal structures. First, differential thermal analysis (DTA) and thermogravimetric analysis (TG) measurements showed the formation of hydrated crystals for C4G and C5G, respectively. Second, the crystal structures of CnG (*n* = 4, 5, 6) in both anhydrous and hydrated states were examined using a temperature-controlled powder X-ray diffraction (PXRD) measurement. Both hydrate and anhydrous crystals of C4G and C5G with critical packing parameters (CPPs) less than 0.33 formed cubic crystal phases. Bilayer lengths, calculated from the main diffraction peaks in each PXRD profile, depended on crystalline moisture for C5G, but no significant change was confirmed for C4G, indicating that the properties of each hydrophilic layer differ. However, C6G with a CPP of 0.42 formed a crystal structure with a modulated lamellar structure similar to C7G and C8G with similar CPP values. Thus, a glycolipid motif concept with a cubic crystal structure was demonstrated.

## 1. Introduction

Amphiphilic compounds are well-known to emerge in various molecular assembled systems such as one-, two- and three-dimensional structures in a liquid crystalline (LC) state [1,2,3,4,5,6,7,8,9,10,11,12]. These are lamellar for one-dimensional, hexagonal columnar for two-dimensional, and cubic for three-dimensional structures, respectively. Ripple, ribbon and tetragonal columnar structures have been reported as intermediate structures [1,2,3]. Recently, the lipid-based LC nanosystems such as lamellarsome, hexosome and cubosome, which are dispersed particles with an internal structure composed of lamellar, hexagonal columnar and cubic phases, respectively, have attracted much attention for biomedical and pharmaceutical applications [13,14,15,16,17,18,19]. In particular, the cubic structure with space group *P*4_3_2 (212), *P*m3n (223), *F*m3n (225) and *F*d3 m (227) is known as a highly stable motif due to the discontinuous internal structure of cubosome [15,19].

Glycolipid or carbohydrate-based surfactant consisting of sugar-moiety and hydrocarbon tails is one of the representative amphiphilic families. To date, various glycolipids LC formation has been studied. The formation of both normal and inverted cubic LC phases, as well as cubosomes consisting of inverted cubic LC phases in the absence and presence of water, has been reported [9,10,11,12,17,18]. However, the formation of the normal or inverted cubic crystal phases of these compounds in pure and hydrated crystal forms has never been reported.

The crystal structure of glycolipid or carbohydrate-based surfactants has been greatly investigated as a good model for glycolipid molecular assemblies in biological systems [20,21,22,23,24,25,26]. Simple lamellar structures have always been found for single-crystal structure analysis and, as a result, the lattice constants of these typical crystals obey the following conditions: *c* >> *a* > *b*, where *c* typically corresponds to bilayer length [20,21,22,23,24,25,26]. However, recently, anhydrous crystals of mono-alkyl β-D glucoside (CnG) such as heptyl (C7G) and octyl *β*-D-glucosides (C8G) were identified as exceptional examples, which showed *c* ≈ *a* >> *b*, using a grazing-incidence wide-angle X-ray diffraction analysis with a two-dimensional detector (2D-GI-WAXD) on perpendicularly aligned CnG films [27]. The large, splayed sugar head group of C7G and C8G made them form a crystal structure with a modulated lamellar structure.

The relationship between the crystal structure and molecular structure has not been clarified. However, the curvature may predict the crystal structure of the molecule similar to the LC case. In the case of the LC state, as the curvature is varied from negative to positive, there is a passage from discontinuous cubic-hexagonal-bicontinuous cubic-lamellar-bicontinuous cubic-hexagonal-discontinuous cubic phases across the phase diagram as a molecular curvature, and the concentration changes [2,3,4,5,6,7,8,9,10,11,12,14].

The critical packing parameter (CPP), defined as

*CPP* = *V*/*AL* (1)

*V* = (27.4 + 26.9 *n*) (2)

**Figure 1:**
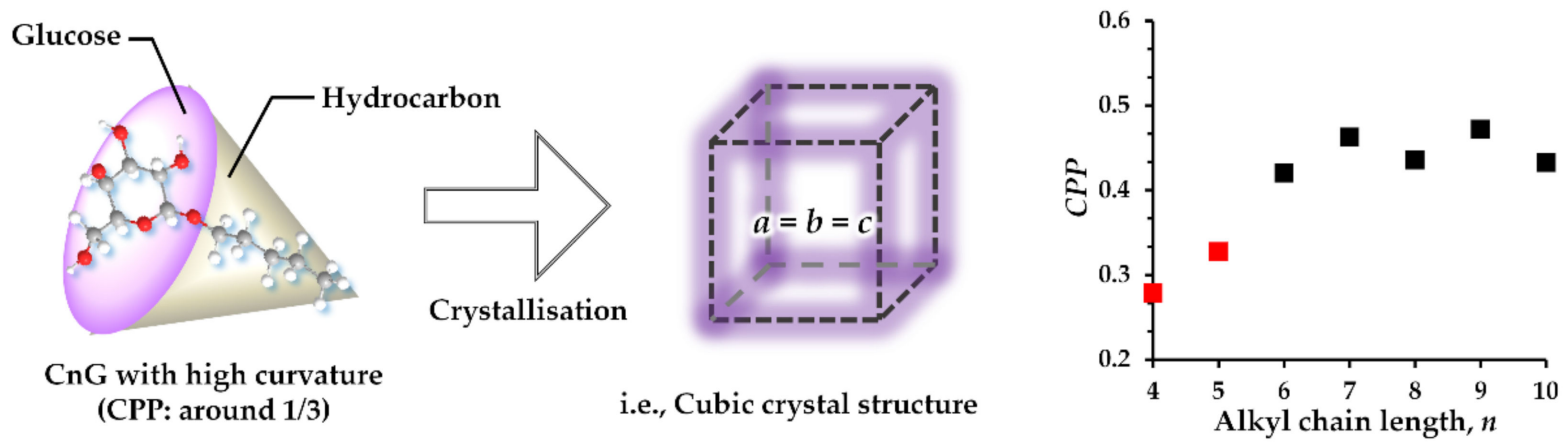
(**Left**). Expected cubic crystal formation from short-chain CnG with low CPP. (**Right**) CPP vs. alkyl chain length, *n*. The CPP of C4G was calculated using the predicted *A* value, which may result in some errors. The red squares were located below 0.33, respectively.

*L* = (1.5 + 1.265 *n*) (3)

where *V* represents the hydrophobic carbon volume, *A* is the cross-sectional area of the hydrophilic head group, *L* is the hydrophobic chain length in a molten state, used here as the relevant value of the curvature [28]. Here, the normal phase anticipated when CPP < 1, while CPP > 1 will give inversed structures. Based on the assumption that *V* = 215.7 and 242.6 Å^3^, *A* = 45.0 and 47.9 Å^2^ [29] and *L* = 10.4 and 11.6 Å, respectively, CPP values of C7G and C8G were calculated as 0.46 and 0.44, respectively. Therefore, they should not form a lamellar structure. However, it was proposed that the strong association between the hydrophobic tails made them into stable interdigitated bilayered structures, which afforded the planar bilayer [10]. Thus, they formed the lamellar LC phase before crystallisation and, as a result, the crystal structure with a lamellar structure formed. However, we expected that the CnG with a shorter alkyl chain with a weak association between hydrocarbon chains and a larger molecular curvature or much smaller CPP is a suitable motif for obtaining alternative crystal structures, such as cubic structures, rather than lamellar structures (Figure 1, left).

To clarify this, we examined the formation of hydrated and anhydrous crystals for short-chain CnG (*n* = 4, 5, 6). The CPP values for C5G and C6G were calculated as 0.33 and 0.42, respectively, based on assumptions of *V* = 161.9 and 188.8 Å^3^, *A* = 63.2 and 49.4 Å^2^ [29] and *L* = 7.83 and 9.09 Å, respectively. The values of *V* and *L* can be calculated as *L* = 6.56 and *V* = 135.0 Å^3^ for C4G using Equations (2) and (3), but the *A* value has not been reported. Therefore, CPP for C4G could not be calculated using Equation (1). However, it was assumed to have a similar or larger value than that of C5G. We predicted the *A* value for C4G using a fitting curve of *n* vs. *A* for CnG (n = 5, 6, 7, 8, 9, 10) [29] and *n* = 4 (Figure S1 and Table S1, Supplementary Materials). Therefore, we obtained a value of 73.8 Å, affording 0.28 as the CPP for C4G (Figure 1, right).

The glass transition and melting behaviour for CnG (n = 4, 5, 6) have previously been investigated [29], but a crystal structural analysis for both hydrate and anhydrous crystals has not been performed. In addition, hydrated crystal formation and its dehydration behaviour have not been investigated in detail [29]. Although preparing single crystals of appropriate sizes for single structure analysis was difficult, we investigated their crystal structures in this study using simultaneous DTA–TG measurement, temperature-controlled PXRD measurement, and 2D-GI-WAXD analysis using powder samples. Hence, the molecular motif concept, which provides a cubic crystal, was proposed for simple amphiphilic sugar molecules.

## 2. Results

### 2.1. Sample Preparation

**Scheme 1:**
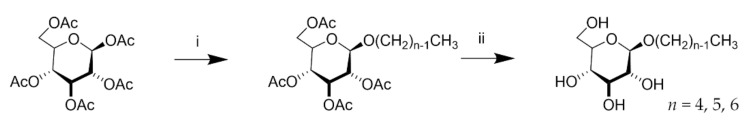
Synthesis of short-chain mono-alkyl β-D-glucoside (CnG; *n* = 4, 5, 6). Reaction conditions (i) 1-Alcohol, BF_3_-Et_2_O/CH_2_Cl_2_ and (ii) NaOMe/MeOH/ion exchange resin, respectively [29].

CnG with alkyl chain lengths ranging from 4 to 6 were prepared, respectively, similarly to the previous study (Scheme 1) [29]. No impurities of alcohol materials or α-anomer by-product were detected in ^1^H-NMR spectra, showing high purity (Figure S2, Supplementary Materials). C6G crystallised in the bulk state at ambient atmosphere, whereas C4G and C5G did not readily crystallise in the bulk state. Therefore, the crystals of C4G and C5G were obtained by precipitating in the acetone/hexane solution mixture. These samples were stored under humidified conditions to make them hydrated crystals. The absence of organic solvents, such as acetone and hexane, was confirmed in the ^1^H-NMR spectra.

### 2.2. Study on Hydrate State

**Figure 2:**
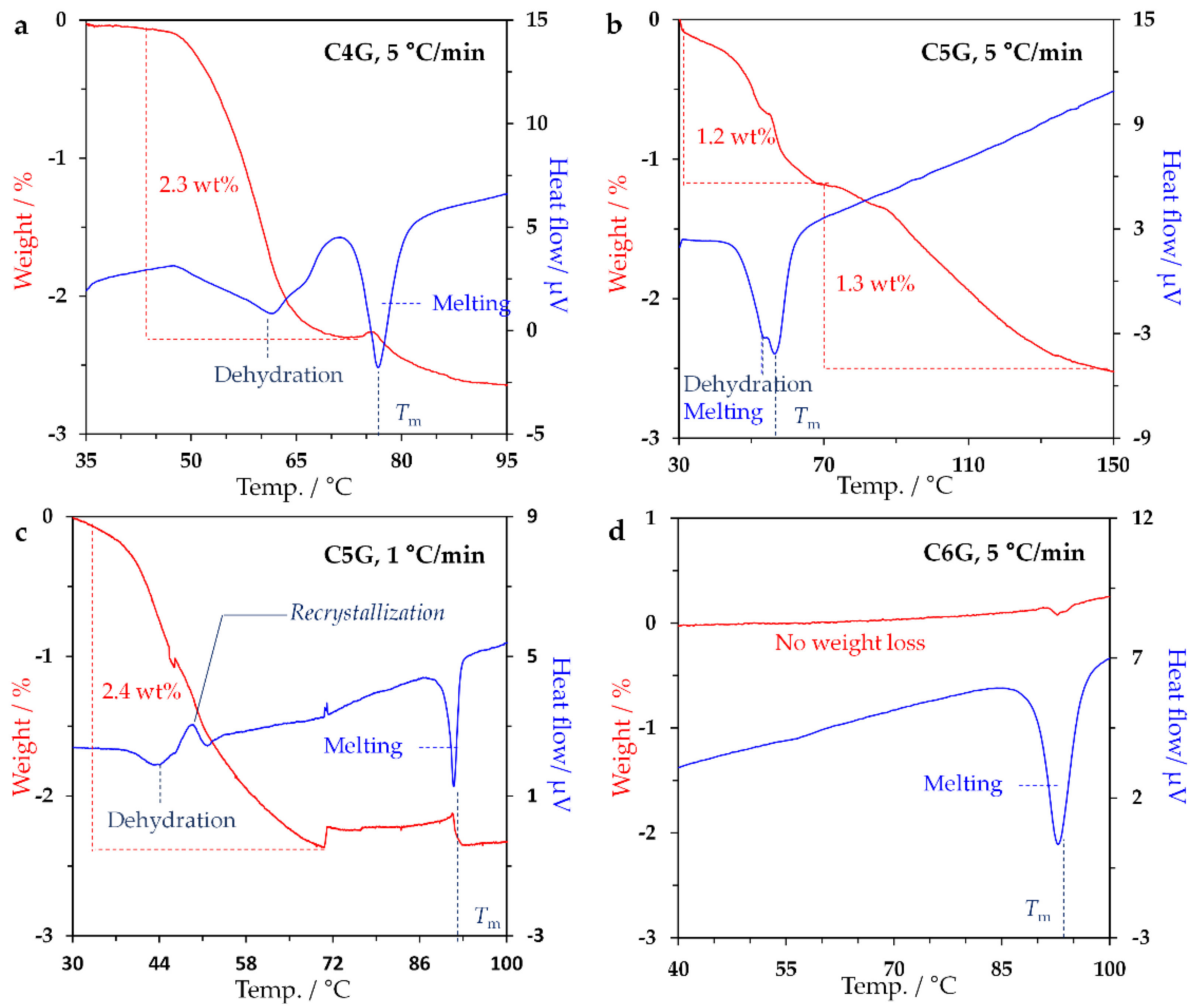
Differential thermal analysis−thermogravimetric analysis simultaneously measurement profiles of (**a**) C4G with the heating rate at 5 °C/min, (**b**) C5G with the heating rate at 5 °C/min, (**c**) C5G with the heating rate at 1 °C/min and (**d**) C6G with the heating rate at 5 °C/min.

First, the hydrated state of each initial CnG (*n* = 4, 5, 6) crystal was analysed using DTA-TG analyses. As shown in Figure 2, each crystal showed different dehydration behaviour. First, the reduction of 2.3-wt% weight loss between 50 °C and 60 °C was confirmed in the heating process of the C4G sample before melting in the DTA–TG profile (Figure 2a). It showed that 0.32 H_2_O hydrated crystal (Cr-h) had formed at the initial state. However, the C5G sample showed the 1.2-wt% weight loss before melting and 1.3-wt% weight loss after melting in the DTA–TG profile obtained at a heating rate of 5 °C/min (Figure 2b). The weight loss was almost the same for heating with the temperature rate of 1 °C/min and 5 °C/min for C5G. Here, the melting temperature was observed around 55 °C. Since the melting point (*T_m_*) of anhydrous C5G is reported to be around 90 °C [29], *T_m_* around 55 °C was attributed to the melting point of 0.32 H_2_O hydrated crystal state (Cr-h1).

However, when we performed the DTA–TG analysis at a 1 °C/min rate, 2.4-wt% weight loss occurred before melting around 90 °C (Figure 2c), indicating that complete dehydration occurred in the crystal state from hydrated crystal. Noteworthy, the sign of recrystallisation was observed as the exothermic peak during the dehydration process, suggesting that the hydrated crystal (Cr-h2) formed at that time. As the dehydration gradually advances in the slow heating condition, the transformation to anhydrous crystals seemed to occur. Contrary to the hydrated crystal formations for C4G and C5G, no dehydration occurred for C6G, suggesting that C6G did not form a hydrated crystal.

Then, temperature dependent PXRD analysis was conducted, considering these hydrated crystal formations and dehydration behaviours, respectively.

### 2.3. Investigation on Phase Transition Behaviour

**Figure 3:**
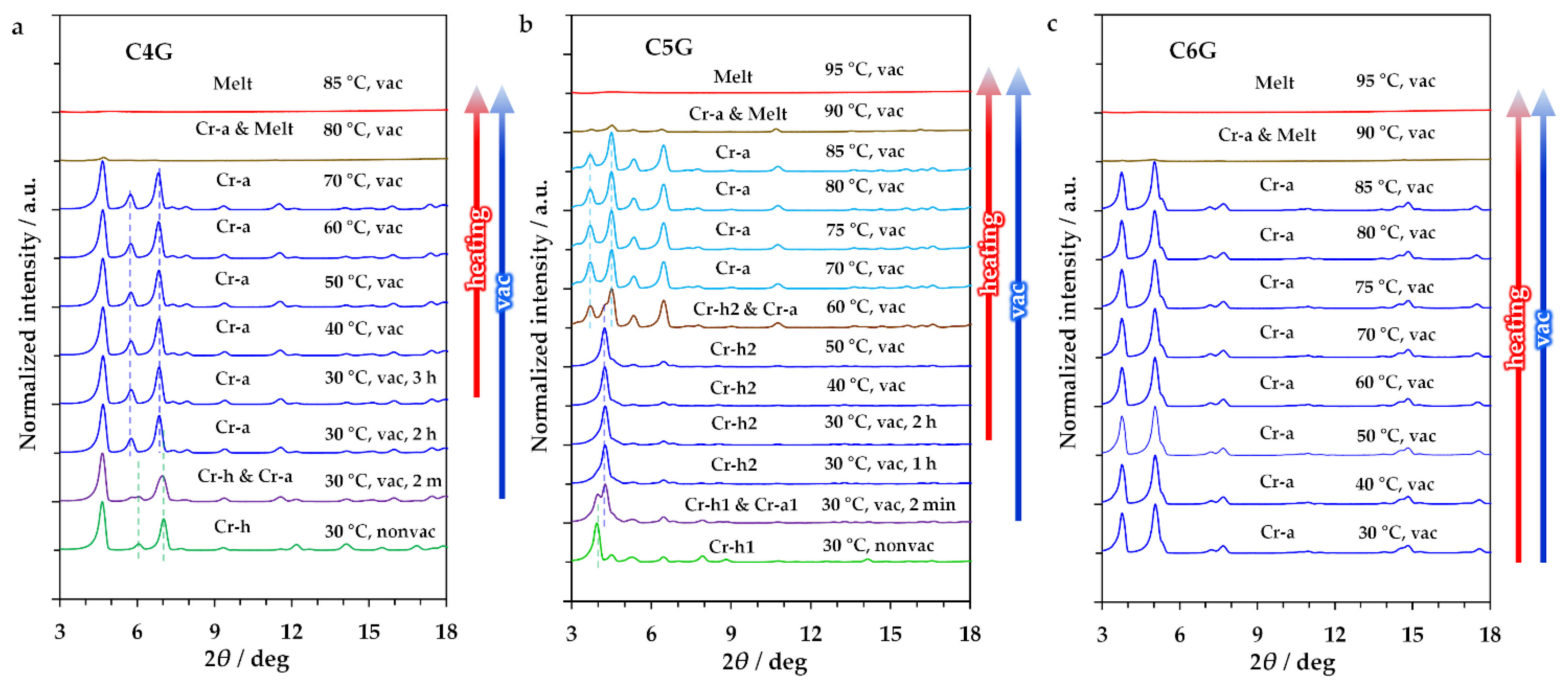
Powder X-ray diffraction profiles of (**a**) C4G, (**b**) C5G, and (**c**) C6G, respectively, under vacuum (vac) and non-vacuum (nonvac) conditions in the heating measurement. “-a” means ‘anhydrous crystal’, and “-h” means ‘hydrated crystal’.

Figure 3a–c shows the PXRD profiles of CnG at various temperatures. The analysis of C4G and C5G started with 0.32 H_2_O hydrated crystals and C6G with an anhydrous crystal state, respectively. Each sample was measured at first under ambient atmosphere and subsequently measured under vacuum conditions at 30 °C. After confirmation that the crystal structure became a thermodynamically stable state under vacuum conditions in the measurement of changes over time, then heating measurements under vacuum conditions were performed.

In the PXRD measurement, the variations in the PXRD profile due to dehydration were confirmed for C4G and C5G (Figure 3a,b). The diffraction patterns for C4G hydrated crystals changed slightly during vacuum drying (i.e., a peak around 6 degrees disappeared [for Cr-h] and an additional one appeared around 5.5 degrees). Changes in the diffraction pattern of C5G were observed for the main diffraction peaks around 4 degrees.

No change in the diffraction patterns for C4G was confirmed in the subsequent heating measurement until it melts at *T_m_* (Figure 3a), indicating that the crystal had already become anhydrous (Cr-a) from hydrated crystal (Cr-h) at 30 °C after vacuum drying. In the case of C5G, which showed two hydrated crystals formations (Cr-h1 and Cr-h2) in the DTA–TG analysis (Figure 2b,c), an additional change was discernible in the diffraction profile when the temperature increased to around 60 °C under vacuum drying (Figure 3b). It means that the transformation from Cr-h2 to anhydrous crystal (Cr-a), which melts around 90 °C, occurred around 60 °C. Conversely, no change in diffraction patterns was seen for the initial C6G crystal (Cr-a1) until it melted around 90 °C (Figure 3c). No hydrated crystal formation was observed for C6G, as observed by DTA–TG analysis. However, polymorphism has previously been reported for C6G [29]; polarized microscopy observation and differential scanning calorimetry (DSC) showed several crystals. Furthermore, C6G exhibited polymorphism when it was recrystallised under vacuum conditions at 50 °C in this study (Figure S3, Supplementary Materials). The obtained crystal seemed to consist of two anhydrous forms (Cr-a1 & Cr-a2), and no pure C6G crystal (a2) PXRD profile could be obtained. Above 70 °C, Cr-a2 transformed into Cr-a1, and only Cr-a1 existed.

**Figure 4:**
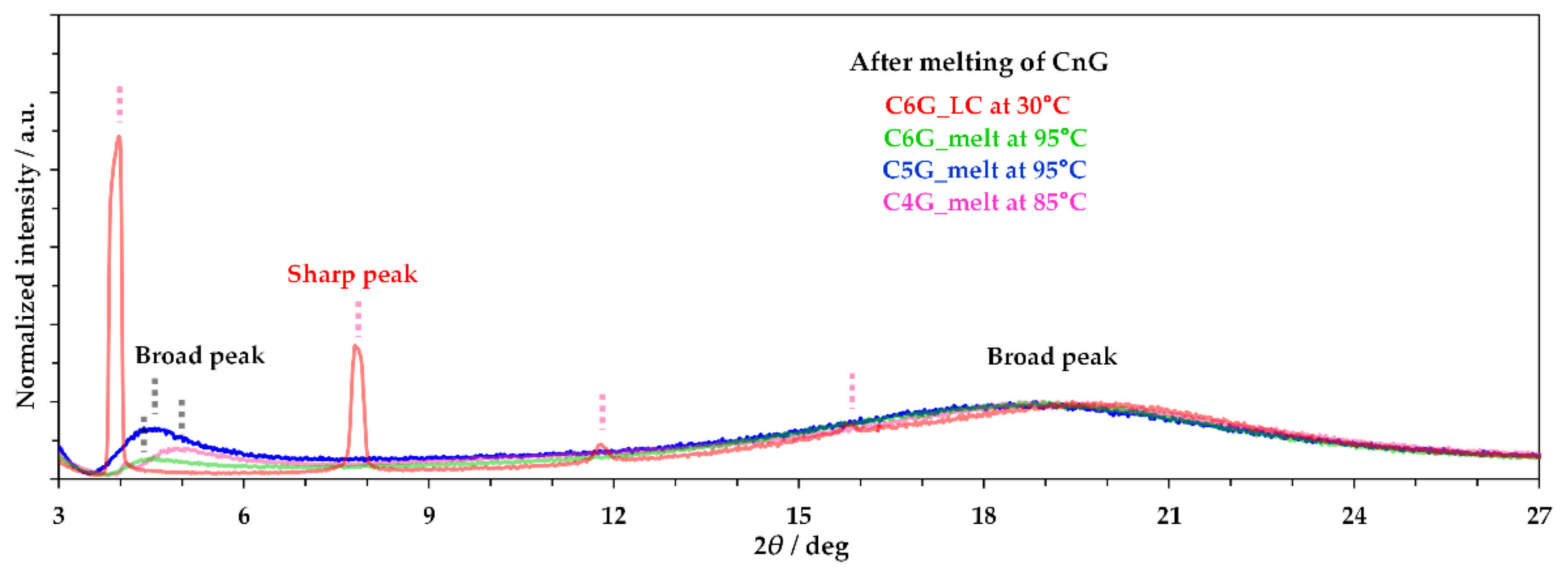
Powder X-ray diffraction profiles of CnG (*n* = 4, 5, 6) at 90 °C and at 30 °C for C6G under vacuum conditions after melting each crystal.

Meanwhile, broad peaks were seen in low diffraction regions of each PXRD profile after each anhydrous crystal was melted (Figure 4 and Table 1). The broad peak shows a slight periodicity corresponding to the bilayer structure. No sharp diffraction peak was observed for the melts of C4G and C5G at room temperature (data not shown). When the C6G melt cooled to room temperature, DSC and PXRD were used to confirm the formation of the lamellar (Lα) LC phase [29]. Monotropical Lα LC phase was conformed using temperature dependent PXRD analysis when the C6G melt cooled below 50 °C, as previously reported (Figure 4 and Figure S4, Supplementary Materials), demonstrating long-range ordering with 1:1/2:1/3:1/4 ratios in the distance based on the calculation using the diffraction peak at 3.98°, 7.80°, 11.9°, and 15.8°, respectively (Table 1). Thus, it was concluded that C4G and C5G did not form lamellar LC phase, whereas C6G formed Lα LC phase at 30 °C after crystallisation.

## 3. Discussion

**Figure 5:**
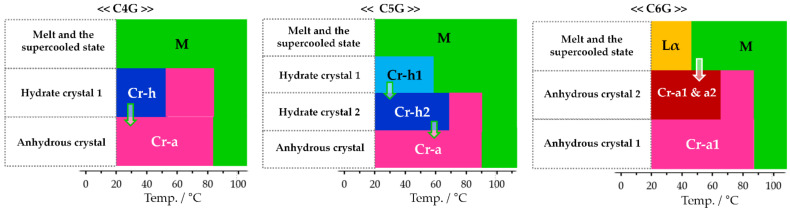
Summary of the phase behaviour of CnG (*n* = 4, 5, 6). Green arrows indicate the phase transition route observed under vacuum conditions at 30 °C. The white arrow indicates the phase transition route under vacuum conditions in the heating process. Lα: Lamellar liquid crystal state. M: Normal micelle. Cr-h: Hydrated crystal. Cr-a: Anhydrous crystal.

Based on the results in Section 2, the dehydration and phase transformation behaviour were summarised as shown in Figure 5. One hydrate and one anhydrous crystal were confirmed for C4G, two hydrates and one anhydrous crystal were confirmed for C5G, and two anhydrous crystals were observed for C6G, respectively. Then, we examined whether the diffraction patterns of these crystals can be assigned as cubic crystals or not. In a nutshell, diffraction patterns were assigned based on the assumption that they formed cubic crystals. The appearance of numerous diffraction peaks at lower diffraction regions indicates that the crystal lattices do not follow *c* >> *a* > *b;* the possibility of simple lamellar crystal structured was denied. Furthermore, since the pure PXRD profile of Cr-a2 of C6G was not obtained, a detailed examination of the sample was avoided. Each experimental data analysed using Rigaku PDXL 2 software are shown in Tables S2–S7, Supplementary Materials.

**Figure 6:**
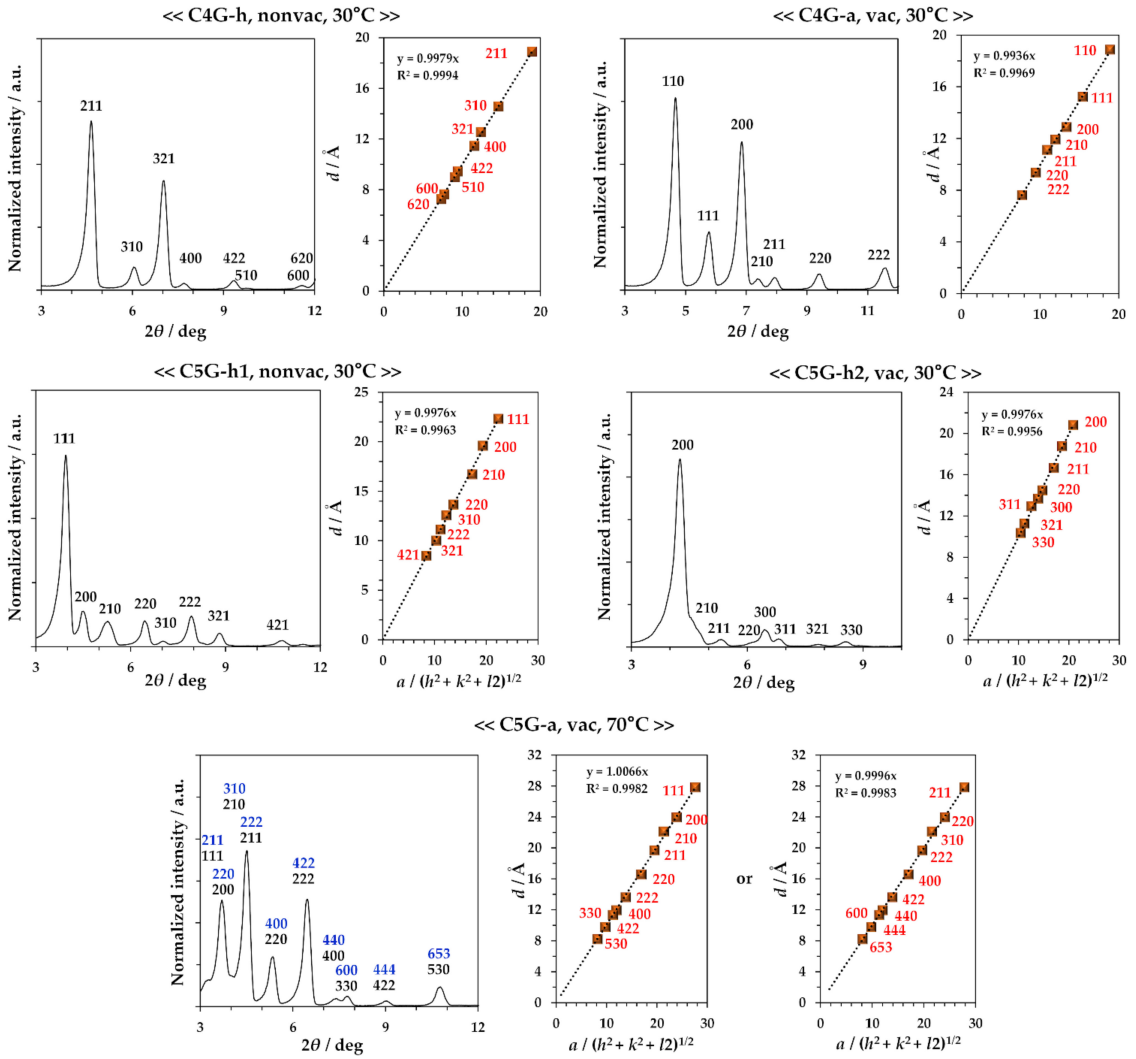
Assignments of diffraction peaks as a cubic phase for hydrate and anhydrous crystals for C4G and C5G, respectively. The left of each sample shows the PXRD profile with a peak numbering, and the right figures show the comparison of experimental and calculated Bragg spacing as the function of *a*/(*h*^2^ + *k*^2^ + *l*^2^)^1/2^ for diffraction peaks numbered.

Figure 6 shows the PXRD profiles with peak numbering and the comparison of experimental and calculated Bragg spacing as the function of *a*/(*h*^2^ + *k*^2^ + *l*^2^)^1/2^ for Cr-h1 and Cr-a for C4G and Cr-h1, Cr-h2 and Cr-a for C5G, respectively. Noteworthy, for these crystals, good coefficients in the relationship of *d_hkl_* vs. *a*/(*h*^2^ + *k*^2^ + *l*^2^)^1/2^ for each crystal state were observed (Figure 6), strongly indicating that this crystal must be cubic. The types of crystal and the lattice constant, *a*, and space group of a cubic crystal are summarised in Table 2. Because C4G and C5G are equipped with high “positive” curvatures, the type of cubic structures should be normal. The space groups were *P*2_1_3 (198), *P*4_2_32 (208), *Pa*-3 (205), or *I*-43*d* (220), respectively (Table 2). Since some undetected reflection may exist, probably due to the low power of commercial X-ray sources compared to synchrotron radiation [30], space groups for some C4G and C5G anhydrous crystals have not been completely determined. Alternatively, because the crystal analysis was performed using the powder state with a diameter of about 300 nm estimated using a Rigaku PDXL 2 software (Tables S2–S6, Supplementary Materials), and the possibility of contamination states of several cubic phases as reported for LC studies [31,32] were not completely denied, especially for the hydrate form. However, the estimates of these space groups differ from *Ia*3*d* for the normal cubic LC phase observed in the C8G/water mixture [33,34], where the continuous water layer was assumed to exist [35]. In this regard, the cubic crystal formed in this study must be formed with strong interactions with sugar-moieties due to low water mole fraction and differs from that observed for the LC structure, which would also lead to an essential difference in physicochemical properties between water-rich cubic LC state and cubic crystal, if they are used as a carrier.

**Figure 7:**
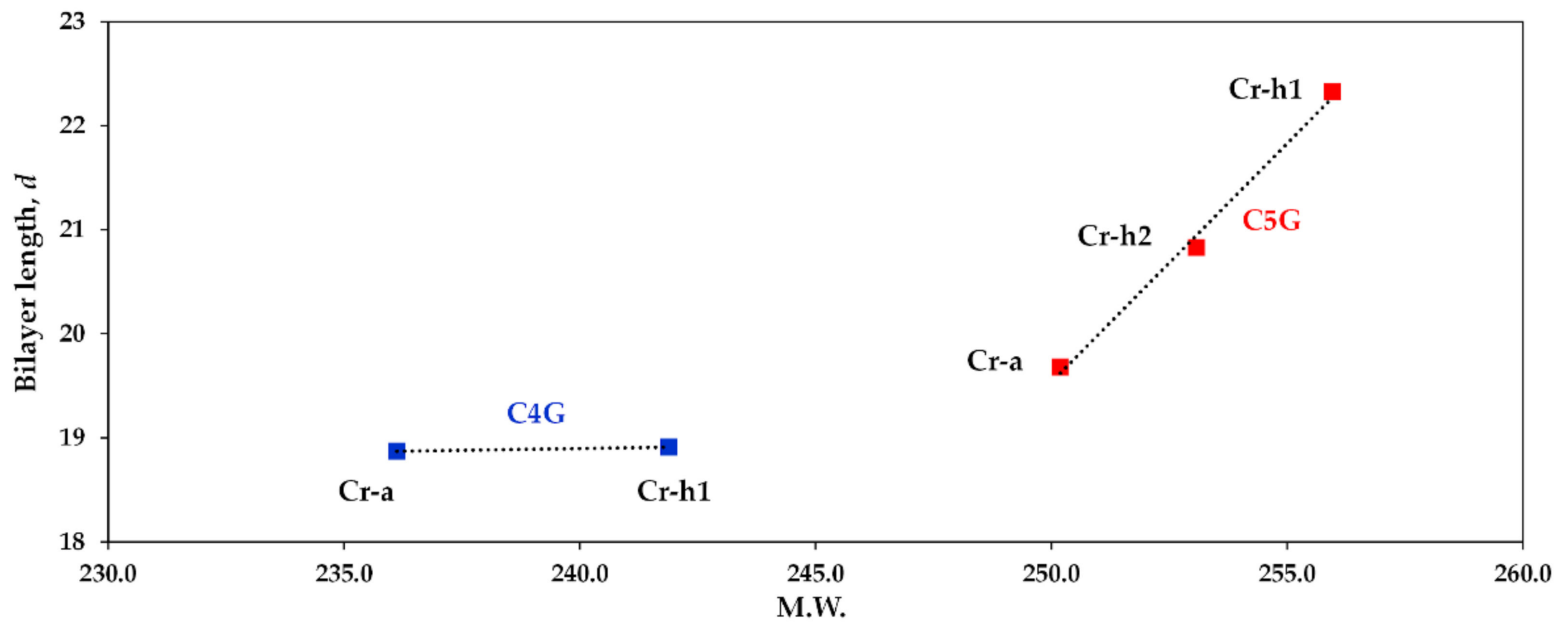
Comparison of bilayer length, *d*, of anhydrous and hydrated crystals for C4G and C5G and molecular weight, M.W.

Notably, a distinct difference was confirmed when we compared the bilayer length, *d*, between anhydrous and hydrated crystals for C4G and C5G, respectively. The data of the hydrated state, molecular weight (M.W.), and *d*, which were determined from the diffraction peak with the highest intensity, were summarized, and the relationship between M.W. and *d* is shown in Table 3 and Figure 7. There was no significant change in *d* for C4G in the presence and absence of hydrate in crystals (Figure 7), indicating that water molecules can be adsorbed in the crystal without distinct changes in the crystal structure. However, the decrease in hydrated water in the crystal reduces *d*, indicating that water molecules can be trapped by interacting with sugar moieties in the crystal, which is accompanied by a significant change in the crystal structure. The rehydration of C4G readily occurred under atmospheric conditions, whereas that of C5G was not readily recognized. The difference in behaviour could be caused by the odd–even effect, thereby altering the intermolecular interaction between sugar moieties in the interfacial region (Figure S1, Supplementary Materials).

On the other hand, no appropriate space group was obtained for anhydrous crystals for C6G, Cr-a1 when the crystal structure was assumed as cubic. However, it formed a relatively aligned crystal film on Si substrate when the C6G solution was spin coated. Therefore, a structural analysis could be performed using a 2D-GI–WAXD analysis (Figure S5, Supplementary Materials). As a result, although full assignment was difficult, unique reflection plots were observed in 2D-GI–WAXD profiles. The profile was similar to that for C7G and C8G, whose crystals were confirmed to be modulated SmC phase in the crystal state [27]. Thus, we assumed that C6G must form a similar structure. These observations are summarised in Table 2, as well as the state before crystallisation.

Noteworthy, a correlation between states before and after crystallisation was observed (Table 2). Cubic crystals were formed when the melt was isotropic; however, they were not formed when the Lα LC phase formed before recrystallisation. The presence of slight periodicity indicated that the isotropic state consists of molecule species interacting with each other at the hydrophilic parts. Recent computer simulations have shown that the isotropic state of hexaoxyethylene dodecyl ether consists of a lamellar-like structure with pores, referred to as an interconnected layer-like pattern [36]. Further investigation is required, but if such three-dimensional bicontinuous structures formed in the isotropic state of C4G and C5G, respectively, the formation of the cubic phase would be reasonable. Based on the present data, the crystal state can be related to the melt structure in the case of CnG. Both a weak association between short hydrocarbon chains and a strong association between sugar-moiety must effectively avoid forming interdigitated flat bilayered structures, which can readily afford a lamellar structure.

## 4. Materials and Methods

### 4.1. Sample Preparation and Characterisation

C4G, C5G and C6G were synthesised according to previous studies, respectively [29]. The purity (>99%) was confirmed by ^1^H-NMR spectroscopy using an FT–NMR spectrometer (ECA-500, JEOL, Tokyo, Japan) and thin layer chromatography–flame ionisation detector (TLC–FID) using an Iatroscan MK-6s with a silica gel rod (LSI Medience Corp. Tokyo, Japan) (acetone: chloroform: methanol: water = 4:9:4:1). ^1^H-NMR spectra (CD_3_OD-*d*_4_) were shown in Figure S2, Supplementary Materials. Film sample of C6G was prepared by spin coating the C6G solution dissolved in 20-wt% methanol/chloroform (2:1 (*v*/*v*)) onto the Si (100) substrate at 4000 rpm for 50 s and subsequent annealing at room temperature in a sealed container, affording crystalline films. It was once heated to 80 °C to obtain a stable anhydrous crystal (Cr-a1) and measured at 30 °C under Ar gas atmosphere.

### 4.2. Simultaneously DTA–TG Measurements

The determination of the dehydration behaviour was also performed under an N_2_ gas flow (100 mL/min) by DTA–TG simultaneously measured using a TG 8120 (Rigaku Corp., Tokyo, Japan). In addition, the number of hydrated H_2_O molecules was estimated by calculating the weight reduction.

### 4.3. X-ray Diffraction Measurements

To investigate the temperature-dependent phase behaviour, PXRD analysis was performed using a multipurpose X-ray diffractometer (SmartLab, Rigaku Corp., Tokyo, Japan) (40 kV, 40 mA). Cu Kα (1.542 Å) was used as the X-ray beam. The powder sample placed in the open glass container was measured under atmospheric conditions at room temperature and under reduced pressure at various temperatures, and out-of-plane (2*θ*/*ω*) scans with parallel beam were performed. The XRD profile and crystal sizes were analysed using the Rigaku PDXL 2 software.

2D-GI–WAXD measurements were performed at BL03XU, SPring-8 (Hyogo, Japan) [27,30,37,38]. Diffraction patterns in the 0–18° range were obtained using an image-plate detector system (R-AXIS IV, Rigaku Corp., Tokyo, Japan) with an incident angle of 0.1° (wavelength = 1.00 Å).

## 5. Conclusions

According to a previous study based on single-crystal structure analysis, many mono-tailed glycolipids have the high formation tendency of a simple lamellar structured crystal [20,21,22,23,24,25,26]. A crystal structure with a modulated lamellar structure was recently proposed for C7G and C8G based on 2D-GI–WAXD analysis [27]. This study showed further unique crystal structures assigned as cubic crystals and intermediate crystals with modulated lamellar structures for CnG with chain lengths from 4 to 6 for both hydrate and anhydrous crystals. C6G formed a crystal state with a modulated lamellar structure similar to C7G and C8G. However, C4G and C5G formed an isotropic state with slightly bilayered ordering before crystallisation, resulting in a cubic crystal. To the best of our knowledge, the cubic crystal formation of simple amphiphilic sugar molecules is unprecedented. Consequently, the significance of the CPP theory for crystalline motifs in the case of weak associations between hydrocarbon chains was proposed. As far as we know, the cubic crystal formation of simple amphiphilic sugar molecules was unprecedented. Consequently, the significance of the CPP theory for crystalline motifs in the case of weak associations between hydrocarbon chains was proposed.

The results of this study will be useful in considering the amphiphilic compounds emerging from cubic lipid crystals, which will be significant for applications that include lipid solid particles because their physical properties and dissolution behaviour will differ from those of LC materials.

## Figures and Tables

**Table 1:** Periodicity observed in melt for CnG (*n* = 4, 5, 6).

CnG	State	Peak Angle [2*θ*]/°	Periodicity, *c*/Å
C4G (85 °C)	Isotropic melt	4.98	18.0
C5G (95 °C)	Isotropic melt	4.56	19.4
C6G (95 °C)	Isotropic melt	4.48	19.7
C6G (30 °C)	Lα LC	3.98, 7.80, 11.9, 15.9	22.2, 11.3, 7.46, 5.59

**Table 2:** Types of crystal and the lattice constant, *a*, and space group of a cubic crystal.

Compound	Crystal Type	State	Crystal Structure Type	Space Group
C4G	0.32 H_2_O Hydrate	Isotropic melt	Cubic crystal with 46.3 Å	*I*-43*d* (220)
C4G	Anhydrous		Cubic crystal with 26.7 Å	*P*2_1_3 (198) or *P*4_2_32 (208)
C5G	0.32 H_2_O Hydrate	Isotropic melt	Cubic crystal with 38.7 Å	*Pa*-3 (205)
C5G	0.16 H_2_O Hydrate		Cubic crystal with 41.7 Å	*I*-43*d* (220)
C5G	Anhydrous		Cubic crystal with 47.8 Å	*Pa*-3 (205) or *I*-43*d* (220)
C6G	Anhydrous	Lα LC	Modulated lamellar structure	Not determined ^2^
C7G ^1^	Anhydrous	Lα LC	Modulated lamellar structure	*P*2_1_
C8G ^1^	Anhydrous	Lα LC	Modulated lamellar structure	*P*2_1_

^1^ The results were cited from ref [27]. ^2^ Owing to the non-clear diffraction profiles, identification of the space group was difficult.

**Table 3:** Comparison of bilayer length, *d*, between anhydrous and hydrated crystals.

CnG	H_2_O	M.W.	2*θ* (Deg)	*d* (Å)
C4G (Cr-h)	0.32	241.9236.1	4.669	18.91
C4G (Cr-a)	0		4.678	18.87
C5G (Cr-h1)	0.32	256.0253.1250.2	3.955	22.33
C5G (Cr-h2)	0.16		4.239	20.83
C5G (Cr-a)	0		4.487	19.68

## Data Availability

Not applicable.

## Supplementary Materials

The following are available online at https://www.mdpi.com/article/10.3390/molecules27144359/s1, Figure S1: Relationship of alkyl chain length, *n* and the occupation area of the molecule at the surface, *A*, for CnG, Figure S2: ^1^H-NMR spectra of (a) C4G, C5G and C6G (solvent; Methanl-*d*_4_), respectively and (b) the corresponding enlarged figures between 4 and 5 ppm, Figure S3: PXRD profiles of C6G in the heating measurement under vacuum conditions, Figure S4: PXRD profiles of C6G in the cooling measurement under vacuum conditions, Figure S5: 2D-WAXD profiles of anhydrous C6G under Ar gas atmosphere, Table S1: A vs. *n* obtained from ref [29], Table S2: Parameters for experimental PXRD profile for Cr h for C4G at 30 °C, Table S3: Parameters for experimental PXRD profile for Cr-a for C4G at 30 °C, Table S4: Parameters for experimental PXRD profile for Cr-h1 for C5G at 30 °C, Table S5: Parameters for experimental PXRD profile for Cr-h2 for C5G at 30 °C. Table S6: Parameters for experimental PXRD profile for Cr-a for C5G at 70 °C. Table S7: Parameters for experimental PXRD profile for Cr-a1 for C6G at 30 °C.

## Sample Availability

Sample of the compound C4G is available from the authors.
