# Supplementary material for: Short-Chain Mono-Alkyl β-D-Glucoside Crystals—Do They Form a Cubic Crystal Structure?
Source: Molecules. 2022 Jul 7;27(14):4359. doi: 10.3390/molecules27144359 (PMC9320782; doi:10.3390/molecules27144359)
Supplement: Supplementary file 1 [file molecules-27-04359-s001.zip › molecules-1771622-supplementary.pdf]

Supplementary Materials

Short-chain mono-alkyl β-D-glucoside crystals: Do they form cubic crystal structure?

Shigesaburo Ogawa [1,*] and Isao Takahashi [2,*]

[1] Department of Food, Aroma and Cosmetic Chemistry, Faculty of Bio-industry, Tokyo University of Agriculture, 196, Yasaka, Hokkaido 099-2493, Japan; so207914@nodai.ac.jp

[2] Department of Physics, School of Science and Technology, Kwansei Gakuin University, Sanda 669-1337, Japan; suikyo@kwansei.ac.jp

* Corresponding author: so207914@nodai.ac.jp (S.O.), suikyo@kwansei.ac.jp (I.T)

Table of Contents

1. Estimation of *A* for C4G....................S2

2. ¹H-NMR spectra of CnG....................S3

3. PXRD measurement of recrystallized C6G....................S4

4. 2D-GI-WAXD measurement of anhydrous C6G crystal....................S6

5. PXRD data of CnG....................S7

1. Estimation of *A* for C4G

Table S1 shows the results of the occupation area of the molecule at the surface, *A*, for CnG (*n* = 5, 6, 7, 8, 9, 10), which was plotted in Figure S1 as a function of alkyl chain length, *n*. For CnG with *n* between 6 and 10, *A* values ranged between 44.3 and 49.4 Å^2. The value showed up-down trends depending on whether *n* is odd or even. That is, the so-called odd–even effect was observed. When *n* is even, *A* values become larger than those obtained for CnG when *n* is odd. However, the *A* of C5G had a significantly higher value compared to other CnG with *n* greater than 6. Furthermore, because n = 4 is even, the *A* of C4G should be larger than that of C5. The fitting curve for CnGs with n between 5 and 10 was obtained (Figure S1). The curve shows that the *A* of C4G reached 73.8 Å^2. We predicted CPP using the calculated *A* for C4G in the main manuscript.

Table S1 *A* v.s. *n* obtained from ref [29]

n	*A* in Å^2
4	Not determined
5	63.2
6	49.4
7	45.0
8	47.9
9	44.3
10	48.4

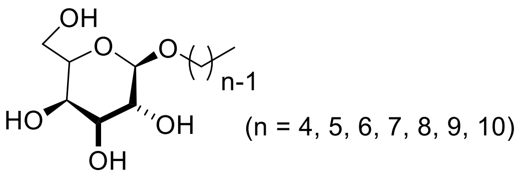

Chemical strcture of **CnG**

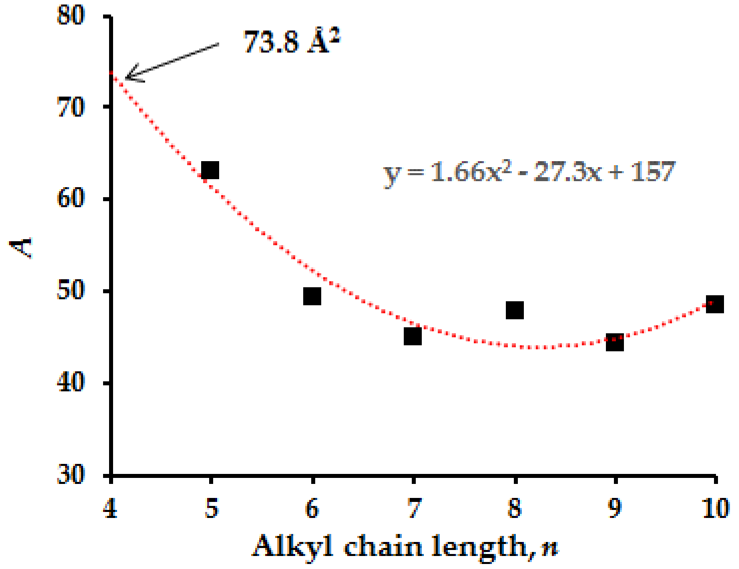

Figure S1. Relationship of alkyl chain length, *n*, and the occupation area of the molecule at the surface, *A*, for CnG. The results of *A* were obtained from ref [29]

2. ^{1}H-NMR spectra of CnG

(a)

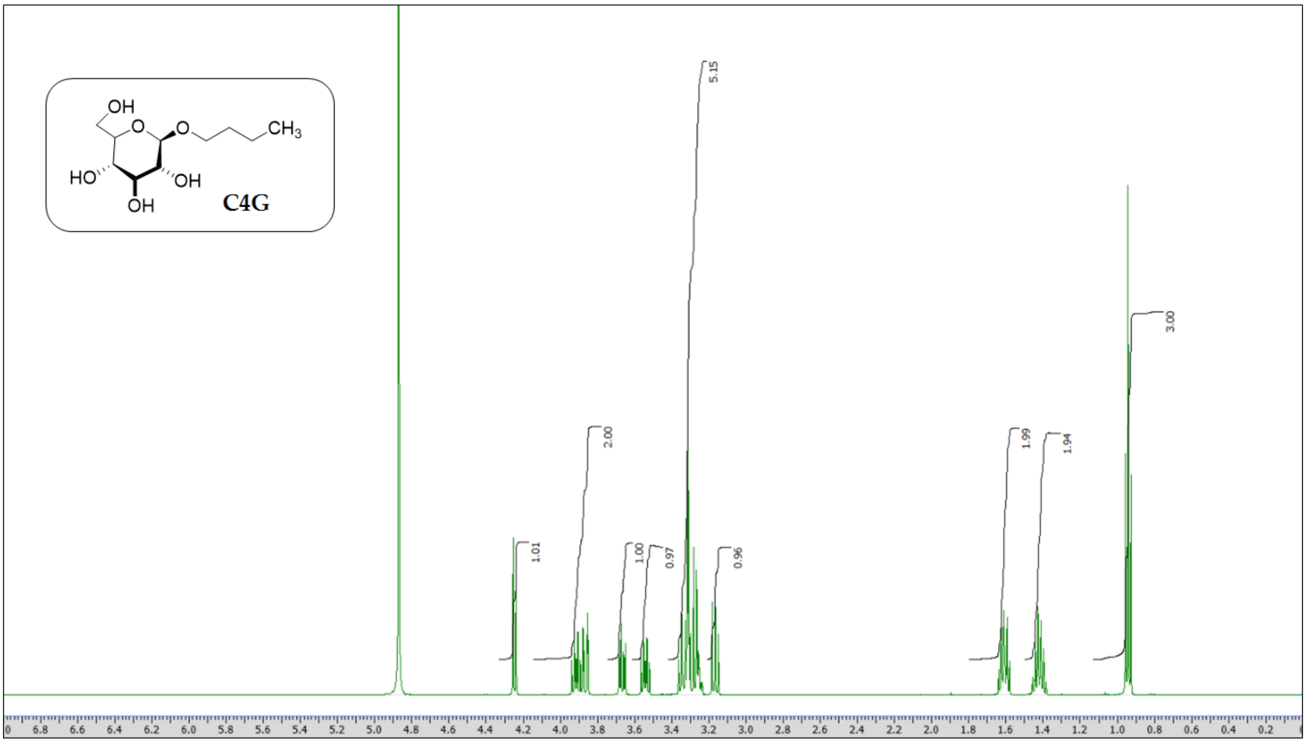

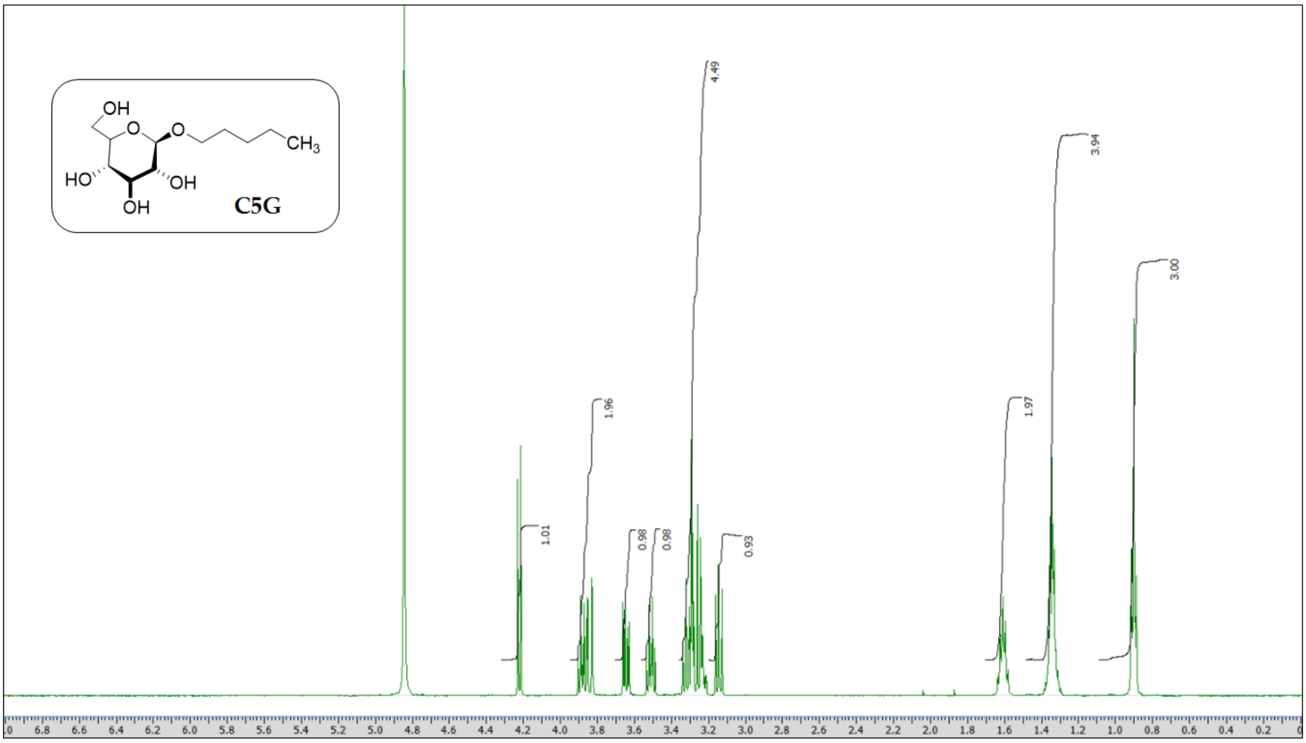

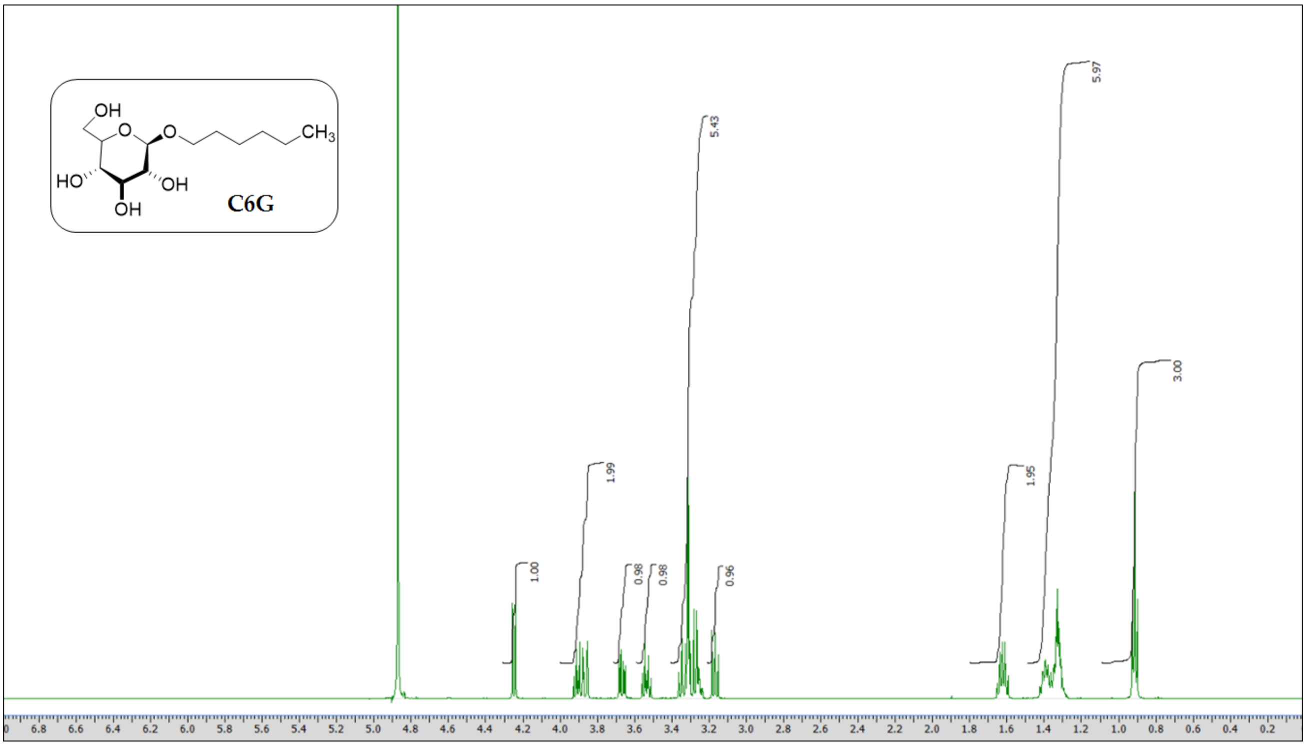

(b)

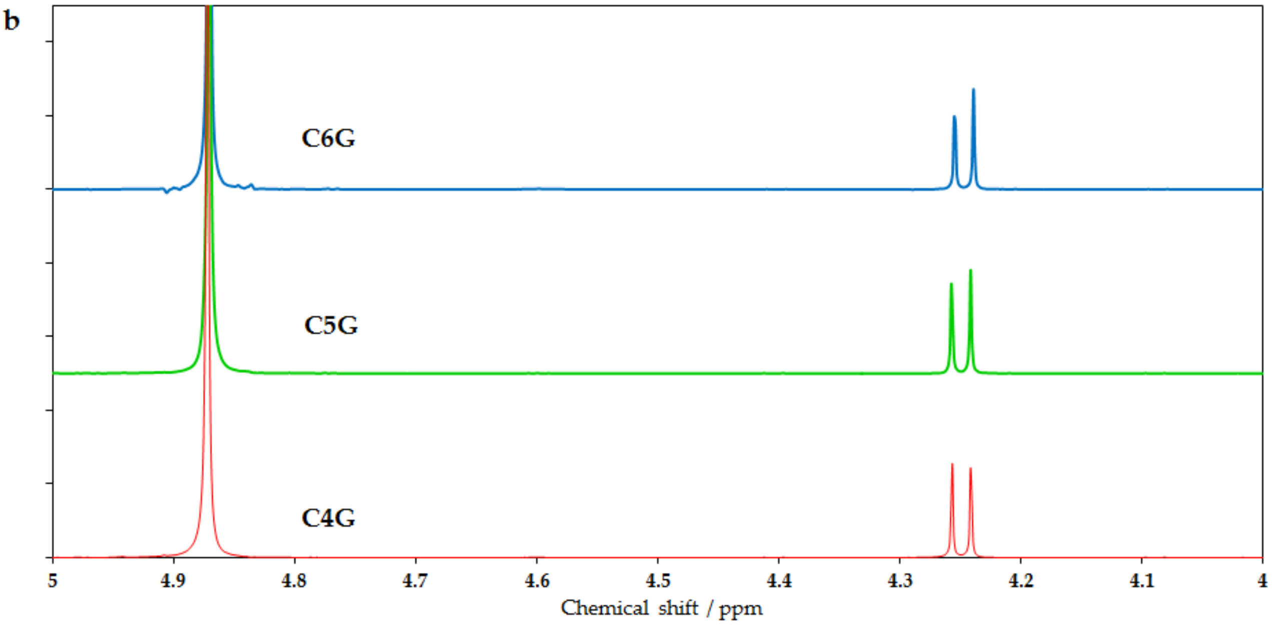

Figure S2. ^{1}H-NMR spectra of (a) C4G, C5G and C6G (solvent; Methanol-d_4), respectively, and (b) the corresponding enlarged figures between 4 and 5 ppm

All ^{1}H-NMR spectra were well-accorded to that of the previous report [29].

3. PXRD measurement of recrystallized C6G

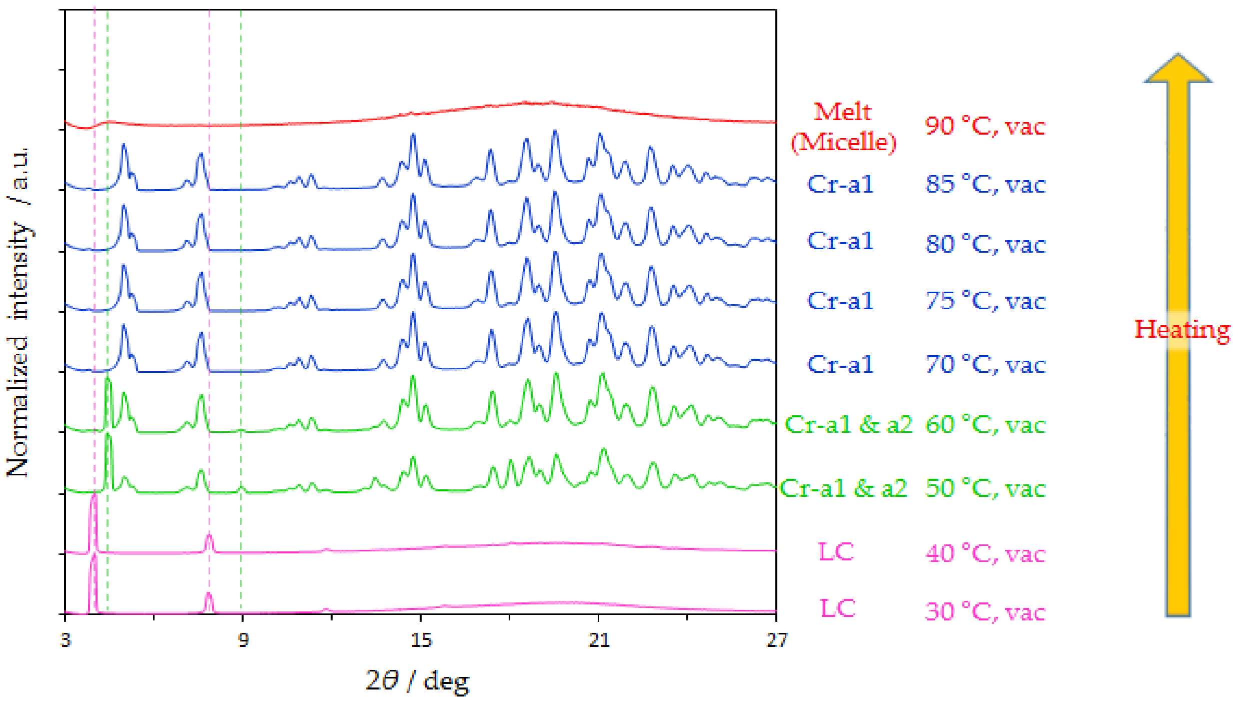

Figure S3. PXRD profiles of C6G in the heating measurement under vacuum conditions. The initial state was a supercooled LC state at 30°C. The diffraction peaks indicated by dotted green lines were assigned to be Cr-a2.

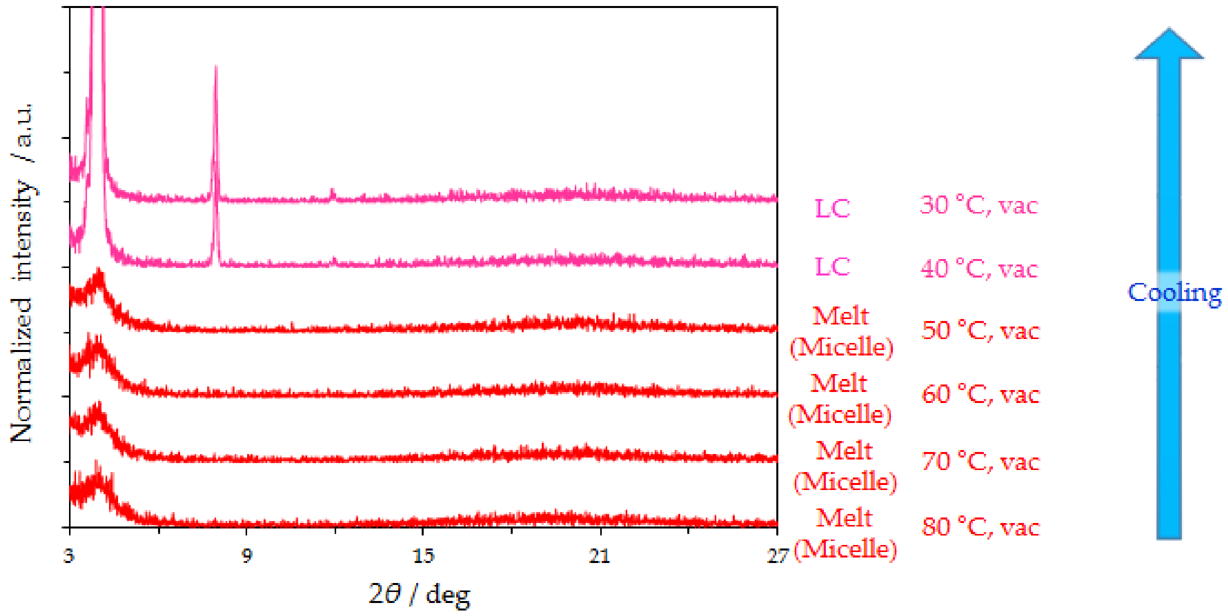

Figure S4. PXRD profiles of C6G in the cooling measurement under vacuum conditions. The initial state was melt state at 80°C.

4. 2D-GI–WAXD measurement of anhydrous C6G crystal

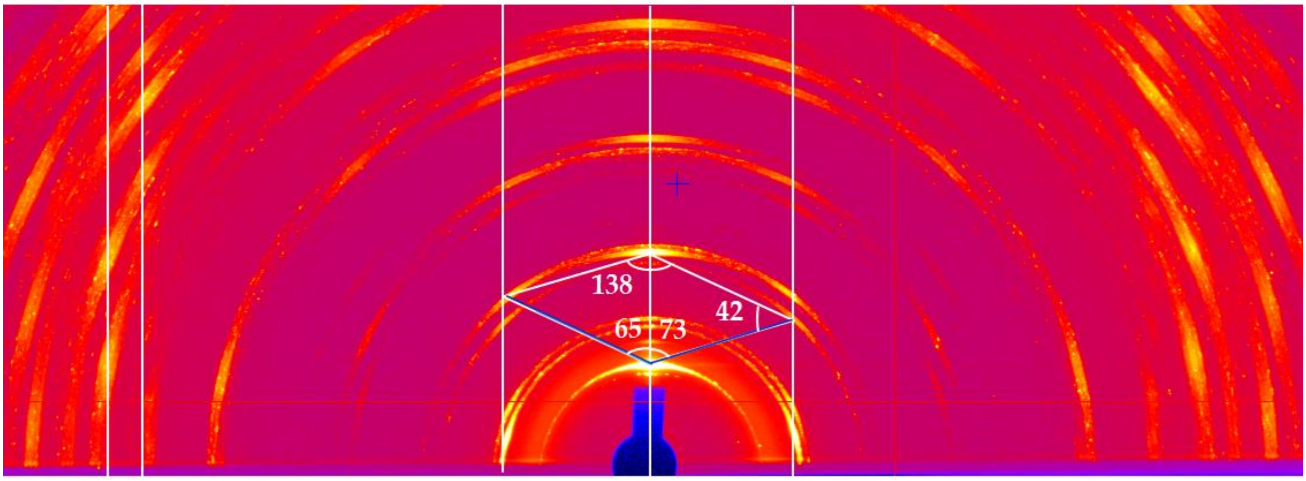

Figure S5. 2D-WAXD profiles of anhydrous C6G (Cr-1) under Ar gas atmosphere.

5. PXRD data of CnG

The parameters such as 2θ, d, height, the full width of half maximum (FWHM), integrated intensity, and crystal size obtained from each PXRD data analyzed using a Rigaku PDXL 2 software were shown in Table S1–S6, respectively.

Table S2 Parameters for experimental PXRD profile for Cr-h for C4G at 30°C

No.	2θ(deg)	d(Å)	Height (cps)	FWHM(deg)	Integrated intensity (cps·deg)	Crystal size(Å)
1	4.669	18.91	30125	0.275	12756	289
2	6.062	14.57	3776	0.276	1478	288
3	7.037	12.552	19652	0.288	7274	277
4	7.712	11.454	1052	0.313	423	255
5	9.353	9.448	1323	0.281	402	283
6	9.821	8.999	66	0.17	12	480
7	11.573	7.64	659	0.37	338	213
8	12.175	7.264	3757	0.282	1449	283
9	12.728	6.95	591	0.31	249	260
10	14.1	6.276	3857	0.347	1607	231
11	14.576	6.072	179	0.18	38	449

12	15.499	5.713	1426	0.258	392	310
13	16.806	5.2712	2508	0.315	841	255
14	17.401	5.092	686	0.23	171	344
15	17.891	4.954	2056	0.64	1408	125
16	18.798	4.7169	2011	0.524	1121	154
17	19.71	4.5	305	0.34	109	240
18	20.08	4.419	615	0.29	190	279
19	20.530	4.3226	3191	0.365	1241	221
20	21.700	4.0921	4061	0.287	1241	282
21	22.04	4.031	448	0.40	189	204
22	22.582	3.9343	2920	0.377	1173	215
23	23.200	3.831	267	0.21	59	392
24	23.644	3.7599	1918	0.282	575	288
25	24.53	3.626	980	0.44	460	184
26	24.949	3.5661	2254	0.375	900	217
27	25.774	3.4538	534	0.258	147	316
28	26.92	3.310	401	0.37	156	224
29	27.338	3.2596	1441	0.33	501	250
30	28.078	3.1754	394	0.28	118	291
31	28.706	3.107	418	0.35	155	236
32	29.799	2.9958	225	0.18	43	463

Table S3 Parameters for experimental PXRD profile for Cr-a for C4G at 30°C.

No.	2θ(deg)	d(Å)	Height (cps)	FWHM(deg)	Integrated intensity (cps·deg)	Crystal size(Å)
1	4.678	18.87	23255	0.277	8514	287
2	5.799	15.23	6575	0.283	1981	281
3	6.853	12.887	20751	0.271	7891	293
4	7.409	11.922	1361	0.20	385	394
5	7.934	11.13	1630	0.51	1174	155
6	9.43	9.37	1845	0.33	917	239
7	11.593	7.627	2751	0.355	1152	225
8	12.216	7.239	677	0.28	224	285
9	14.139	6.2588	894	0.262	266	305
10	15.096	5.864	735	0.400	333	200
11	15.979	5.542	1783	0.323	654	248
12	16.79	5.277	438	0.93	461	87

13	17.417	5.0877	2055	0.274	640	293
14	17.974	4.9313	2381	0.733	1979	110
15	18.945	4.6806	1505	0.341	582	236
16	20.060	4.4228	960	0.251	257	321
17	20.653	4.2971	2487	0.446	1181	181
18	21.525	4.1249	1498	0.311	496	260
19	21.937	4.049	579	0.37	228	218
20	22.400	3.9659	1304	0.317	439	256
21	23.192	3.8321	1983	0.386	814	210
22	23.976	3.7085	1333	0.318	451	256
23	25.134	3.5403	1153	0.541	664	151
24	26.607	3.3475	1155	0.323	397	253
25	27.55	3.235	171	0.40	73	203
26	28.035	3.1802	328	0.24	84	340
27	28.66	3.112	306	0.22	73	366
28	28.93	3.084	342	0.31	111	269
29	29.688	3.0068	221	0.21	49	397

Table S4 Parameters for experimental PXRD profile for Cr-h1 for C5G at 30°C.

No.	2θ(deg)	d(Å)	Height (cps)	FWHM(deg)	Integrated intensity (cps·deg)	Crystal size(Å)
1	3.955	22.325	41485	0.268	15624	297
2	4.506	19.594	7524	0.270	2854	295
3	5.284	16.71	5447	0.388	2970	205
4	6.462	13.667	5259	0.250	1631	318
5	7.034	12.557	706	0.188	164	424
6	7.939	11.127	6130	0.277	2082	287
7	8.811	10.028	2585	0.262	766	304
8	10.44	8.47	161	0.46	85	172
9	10.782	8.199	1150	0.345	454	231
10	11.428	7.737	341	0.317	124	251
11	11.932	7.411	156	0.25	45	316
12	12.586	7.027	767	0.265	232	301
13	12.98	6.815	324	0.193	71	415
14	13.7	6.458	419	0.246	110	325
15	14.149	6.2543	3035	0.253	817	316
16	14.811	5.976	522	0.315	175	254

17	15.671	5.65	730	0.254	197	316
18	16.125	5.492	523	0.46	254	176
19	16.557	5.35	1111	0.232	274	346
20	17.97	4.932	688	0.536	392	150
21	18.902	4.6911	1660	0.354	625	228
22	19.762	4.4889	1646	0.262	459	308
23	20.421	4.3454	3290	0.336	1176	240
24	21.169	4.194	905	0.29	283	275
25	21.537	4.1227	4002	0.264	1124	307
26	21.883	4.0583	891	0.166	158	486
27	22.670	3.919	1493	0.30	482	267
28	23.104	3.8465	1431	0.30	450	274
29	23.54	3.777	309	0.29	95	282
30	24.557	3.6222	537	0.207	119	392
31	25.115	3.543	871	0.34	313	241
32	25.45	3.496	592	0.30	187	275
33	26.39	3.374	285	0.36	109	227
34	27.312	3.2627	461	0.22	109	369
35	27.710	3.2167	428	0.33	148	251
36	28.381	3.142	465	0.279	138	294
37	29.43	3.032	271	0.26	75	317

Table S5 Parameters for experimental PXRD profile for Cr-h2 for C5G at 30°C.

No.	2θ(deg)	d(Å)	Height (cps)	FWHM(deg)	Integrated intensity (cps·deg)	Crystal size(Å)
1	4.239	20.83	48086	0.270	19326	295
2	4.713	18.75	Peak was too small to be detected and manually examined			
3	5.303	16.65	1924	0.24	699	326
4	6.1	14.49	Peak was too small to be detected and manually examined			
5	6.454	13.683	4304	0.257	1754	310
6	6.822	12.947	1873	0.241	715	330
7	7.838	11.271	550	0.321	238	248
8	8.537	10.35	1189	0.263	422	302
9	8.89	9.94	252	0.42	143	189
10	10.007	8.832	132	0.22	31	357
11	10.765	8.212	599	0.316	201	253
12	11.545	7.659	152	0.26	43	303

13	12.341	7.166	355	0.62	235	128
14	12.877	6.869	900	0.268	257	298
15	13.271	6.666	1443	0.267	410	299
16	13.698	6.459	774	0.282	232	284
17	14.165	6.247	493	0.344	181	232
18	15.701	5.64	207	0.20	44(4)	406
19	16.171	5.4767	758	0.281	227	286
20	16.631	5.3263	986	0.238	249	338
21	17.788	4.982	414	0.56	249	142
22	18.729	4.734	716	0.20	153	401
23	18.92	4.686	620	0.26	174	305
24	19.85	4.47	547	0.30	177	265
25	20.19	4.396	729	0.48	375	167
26	20.619	4.3041	686	0.24	172	342
27	21.059	4.2151	1454	0.410	635	197
28	22.089	4.0209	884	0.350	330	231
29	22.53	3.94	308	0.74	243	109
30	23.658	3.7577	583	0.238	148	341
31	24.358	3.6512	378	0.34	137	239
32	24.862	3.5784	382	0.254	103	320
33	25.460	3.4956	266	0.21	59	393
34	27.2	3.276	86	0.31	53	263
35	28.167	3.1656	199	0.46	102	179

Table S6 Parameters for experimental PXRD profile for Cr-a for C5G at 70°C.

No.	2θ(deg)	d(Å)	Height (cps)	FWHM(deg)	Integrated intensity (cps·deg)	Crystal size(Å)
1	3.17	27.82	4215	0.26	1298.24	306
2	3.69	23.95	12215	0.32	5721	247
3	3.99	22.15	Peak was too small to be detected and manually examined			
4	4.487	19.68	20844	0.25	7531	320
5	5.33	16.56	6527	0.24	2259	334
6	6.477	13.635	14829	0.270	5353	294
7	7.392	11.95	1058	0.49	696	161
8	7.777	11.359	1270	0.23	390	346
9	9.038	9.777	529	0.28	167	280
10	10.797	8.187	2295	0.324	851	24

11	11.5046	7.68541	51.3144	0.260	14.4	307
12	11.8817	7.44233	70.4644	0.260	19.8	307
13	12.78	6.919	52	0.22	13	365
14	13.635	6.489	703	0.255	203	314
15	14.006	6.318	266	0.26	78	308
16	14.652	6.041	555	0.225	142	356
17	15.34	5.77	213	0.33	75	247
18	15.623	5.667	969	0.24	256	328
19	16.176	5.475	856	0.267	246	301
20	16.588	5.34	1446	0.250	391	321
21	17.504	5.062	322	0.39	134	205
22	18.085	4.901	1251	0.389	518	207
23	18.779	4.722	568	0.25	148	328
24	19.188	4.622	625	0.32	214	250
25	20.000	4.4359	1298	0.244	338	330
26	20.473	4.335	539	0.21	121	381
27	21.009	4.2252	1412	0.200	300	405
28	21.30	4.168	751	0.35	282	229
29	22.133	4.013	400	0.20	86	400
30	22.625	3.93	593	0.327	207	247
31	23.134	3.8417	376	0.19	77	423
32	23.548	3.7751	1021	0.199	217	407
33	24.05	3.698	136	0.12	17	680
34	24.742	3.5955	488	0.236	123	344
35	25.395	3.5044	688	0.255	187	319
36	26.11	3.41	297	0.40	128	203
37	27.114	3.286	86	0.13	12	621
38	27.584	3.2312	434	0.41	190	199
39	28.256	3.1558	656	0.266	186	307

Table S7 Parameters for experimental PXRD profile for Cr-a1 for C6G at 30°C.

No.	2θ(deg)	d(Å)	Height (cps)	FWHM(deg)	Integrated intensity (cps·deg)	Crystal size(Å)
1	3.77	23.39	20284	0.25	7163	324
2	5.019	17.591	26793	0.307	10774	259
3	7.201	12.27	1495	0.27	537	290
4	7.668	11.519	4058	0.287	1521	278

5	10.158	8.70	68	0.287	23.6	278
6	10.61	8.33	462	0.58	305	137
7	10.643	8.306	171	0.29	59.7	278
8	11.001	8.036	925	0.32	339	246
9	11.476	7.705	379	0.28	120	285
10	11.99	7.38	83	0.43	41	186
11	13.889	6.371	513	0.265	165	302
12	14.534	6.09	2309	0.29	800	281
13	14.821	5.9723	4261	0.256	1329	312
14	15.292	5.79	658	0.41	328	195
15	17.078	5.188	268	0.35	98	233
16	17.577	5.0417	2116	0.277	624	290
17	18.697	4.7421	2297	0.416	1025	194
18	19.165	4.6274	573	0.205	126	392
19	19.662	4.5115	2641	0.402	1139	200
20	20.833	4.2604	925	0.228	225	354
21	21.327	4.1629	2591	0.561	1547	144
22	22.067	4.0249	1229	0.334	436	243
23	22.937	3.8741	1887	0.302	606	269
24	23.678	3.7546	1051	0.282	315	288
25	24.223	3.6713	1192	0.415	527	196
26	24.885	3.575	500	0.24	127	340
27	25.211	3.5296	641	0.28	192	289
28	26.304	3.3854	319	0.18	61	456
29	26.657	3.3413	583	0.39	245	207
30	27.16	3.281	147	0.20	31	413
31	27.839	3.202	396	0.22	94	366
32	28.28	3.153	317	0.33	112	247
33	29.28	3.048	123	0.24	31	341
34	29.659	3.0096	355	0.28	105	297
